# Lisocabtagene maraleucel for treatment of relapsed and refractory primary mediastinal large B‐cell lymphoma in an adolescent patient

**DOI:** 10.1002/jha2.859

**Published:** 2024-02-12

**Authors:** Dasom Lee, Anmol Goyal, William L Wang, Snegha Ananth, Eric Lau, Michael S Binkley, Sushma Bharadwaj, Saurabh Dahiya

**Affiliations:** ^1^ Division of Hematology Stanford University School of Medicine Stanford California USA; ^2^ Division of Blood and Marrow Transplantation and Cellular Therapy Stanford University School of Medicine Stanford California USA; ^3^ Department of Hematology and Oncology Palo Alto Foundation Medical Group Palo Alto California USA; ^4^ Department of Radiation Oncology Stanford University School of Medicine Stanford California USA

**Keywords:** adolescent, AYA, lisocabtagene maraleucel, outcome, primary mediastinal large B‐cell lymphoma, refractory, relapsed, safety

## Abstract

The safety and efficacy of CAR T‐cell therapy are unknown in pediatric and adolescent patients with relapsed or refractory primary mediastinal large B‐cell lymphoma (R/R PMBCL) which is associated with dismal prognosis. Here, we present a case report of a 16‐year‐old patient with R/R PMBCL treated with lisocabtagene maraleucel including correlative studies. Patient achieved complete response at 6 months without cytokine release syndrome and immune effector cell‐associated neurotoxicity syndrome. She only experienced mild cytopenias, requiring filgrastim once. This report highlights the safety and efficacy of lisocabtagene maraleucel in this population, warranting prospective studies to improve clinical outcomes.

**List of Abbreviations**
axi‐cel: axicabtagene ciloleucel
CAR‐T: CD19‐targeted CAR T‐cell therapy
CR: complete response
CRS: cytokine release syndrome
HCT: hematopoietic stem cell
ICANS: immune effector cell‐associated neurotoxicity syndrome
LBCL: large B‐cell lymphoma
liso‐cel: lisocabtagene maraleucel
NHL: non‐Hodgkin's lymphoma
PMBCL: primary mediastinal large B‐cell lymphoma
R/R: relapsed or refractory
US FDA: United States Food and Drug Administration

## 1 INTRODUCTION

The incidence of non‐Hodgkin lymphoma (NHL) in adolescents occurs about 1.8–7.2 per 100,000 per year [1], and one of the common types of NHL in this population is primary mediastinal large B‐cell lymphoma (PMBCL). Upfront treatment with DA‐EPOCH‐R (etoposide, prednisone, vincristine, cyclophosphamide, and doxorubicin with rituximab) as well as programmed death ligand inhibitor have improved 5‐year event‐free survival and overall survival [2, 3]. However, for approximately 10%–30% of patients with refractory or relapsed (R/R) disease, the prognosis is dismal [4], highlighting the need for novel strategies to improve clinical outcomes in these patients. Here, we present a case of a 16‐year‐old patient who received lisocabtagene maraleucel (liso‐cel), CD19‐targeted CAR T‐cell (CAR‐T), for treatment of R/R PMBCL.

## 2 CASE PRESENTATION

A 16‐year‐old female patient was referred to the clinic for treatment of R/R PMBCL. At the age of 15, the patient was first diagnosed with PMBCL. It predominantly presented as anterior mediastinal mass with mass effect on the heart, thoracic aorta, and superior vena cava and generalized lymphadenopathy. The biopsy of the cervical node confirmed histological diagnosis of PMBCL as it showed weakly positive CD30+ and CD15+ B‐cells (Ki67 of 70%–80%) with the presence of MYC rearrangement and molecular Lymph3Cx/PM3CX analysis indicating the PMBCL‐like signature with a probability score of 1.00. She received 6 cycles of DA‐EPOCH‐R as frontline therapy and achieved complete response (CR). Unfortunately, within 7 months after achieving CR, the patient developed relapsed disease confirmed by pericardiocentesis and renal mass biopsy. It involved mediastinal mass, anterior precardiac mass with large pericardial effusion, bilateral renal masses, bilateral pleural effusions, ascites, and retroperitoneal adenopathy. She subsequently received 4 cycles of ICE (ifosfamide, carboplatin, and etoposide) plus pembrolizumab followed by 2 cycles of R‐CyM (cyclophosphamide and melphalan) plus pembrolizumab which resulted in persistent residual disease. Repeat biopsy of the mediastinal mass confirmed the residual disease with CD19 expression on immunohistochemistry (strong, > 95%). CAR‐T is not currently approved for the treatment of PMBCL in the pediatric and adolescent population per the United States Food and Drug Administration (US FDA) approval. However, given such aggressive nature of the disease, she was referred to a bone marrow transplant and cellular therapy clinic for evaluation of potential cellular therapy. Upon discussion with the patient and assenting parent, the decision was made to proceed with CAR‐T, specifically liso‐cel. Bristol Myers Squibb, the manufacturer of the product, approved its use in this setting, and it was financially approved by her insurance provider. At the time of CAR‐T evaluation, CT scan of chest, abdomen, and pelvis with contrast showed mediastinum (7.7 × 3.7 cm) increasing in size and a 2 cm lesion in the right kidney concerning lymphomatous involvement. Lactate dehydrogenase was 515 u/L and C‐reactive protein was 0.4 mg/dL. The patient received radiation therapy (15 Gy in 10 fractions) to the mediastinum as bridging therapy followed by fludarabine (30 mg/m^2^) and cyclophosphamide (300 mg/m^2^) as lymphodepleting chemotherapy.

**FIGURE 1:**
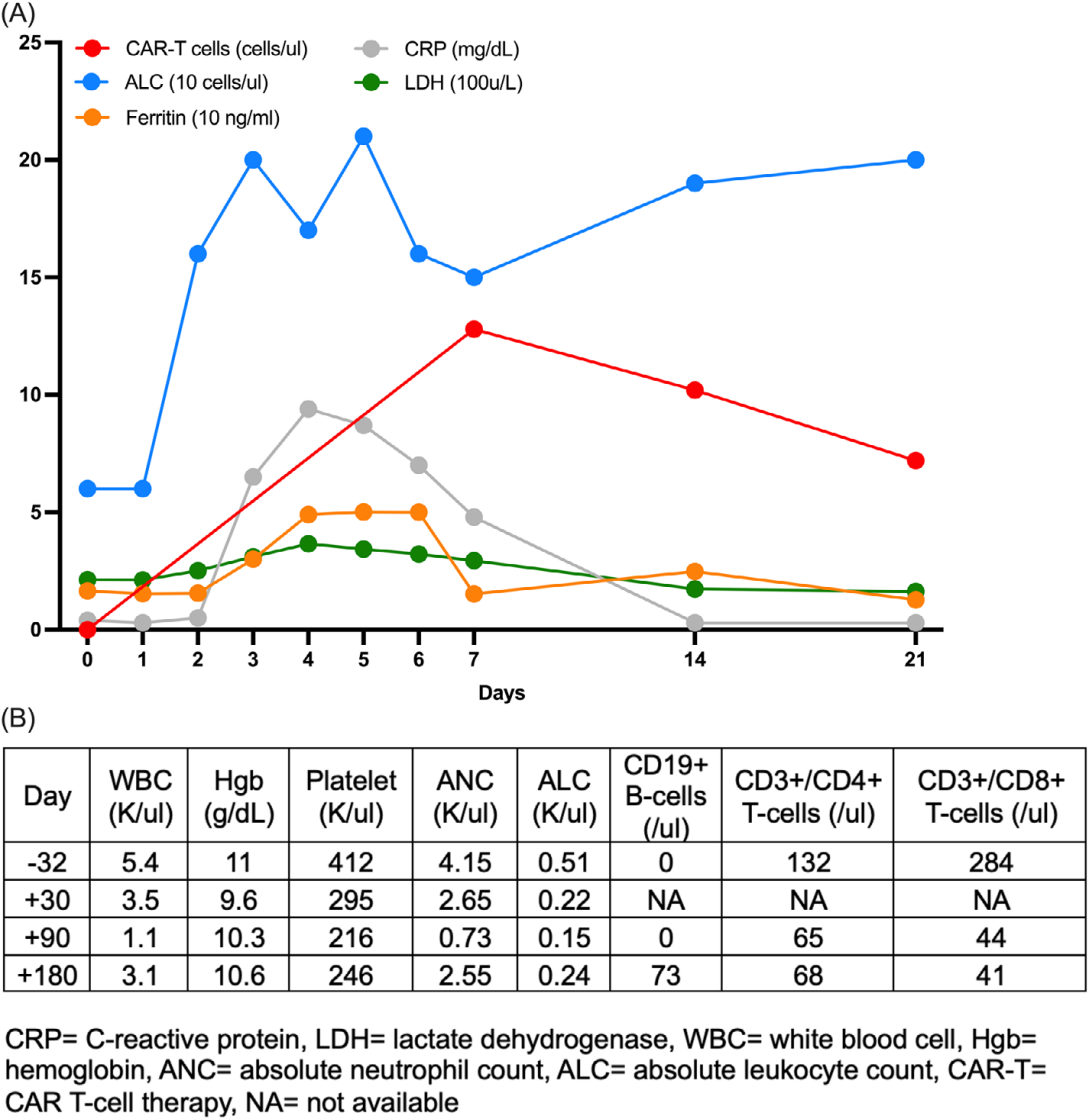
A graph (A) demonstrating inflammation markers, absolute lymphocyte count, and CD19 targeted CAR T‐cell (CAR T)‐cells measured via flow cytometry within 30 days post‐CAR‐T infusion. A table (B) assessing cytopenias and B‐cell aplasia at days +30, +90, and +180 post CAR‐T infusion. The patient received filgrastim for 3 days at day+89.

The patient received in‐specification liso‐cel composed of CD8+ CD3+ cells (dose: 50 × 10^6^ cells/kg) and CD4+ CD3+ cells (dose: 50 × 10^6^ cells/kg) per the FDA‐approved label. No immediate complication after the infusion was reported. In the subsequent 7 days of required observation per our institutional protocol, the patient did not develop any cytokine release syndrome (CRS), immune effector cell‐associated neurotoxicity syndrome (ICANS), hemophagocytic lymphohistocytosis, or medical complication requiring intervention. Her immune effector cell encephalopathy score remained 10 out of 10 throughout the hospitalization. The patient's inflammation markers, absolute lymphocyte count, and CAR T‐cell count measured by flow cytometry are illustrated in Figure 1A. The patient was clinically stable and discharged home after completion of the required 7‐day observation.

**FIGURE 2:**
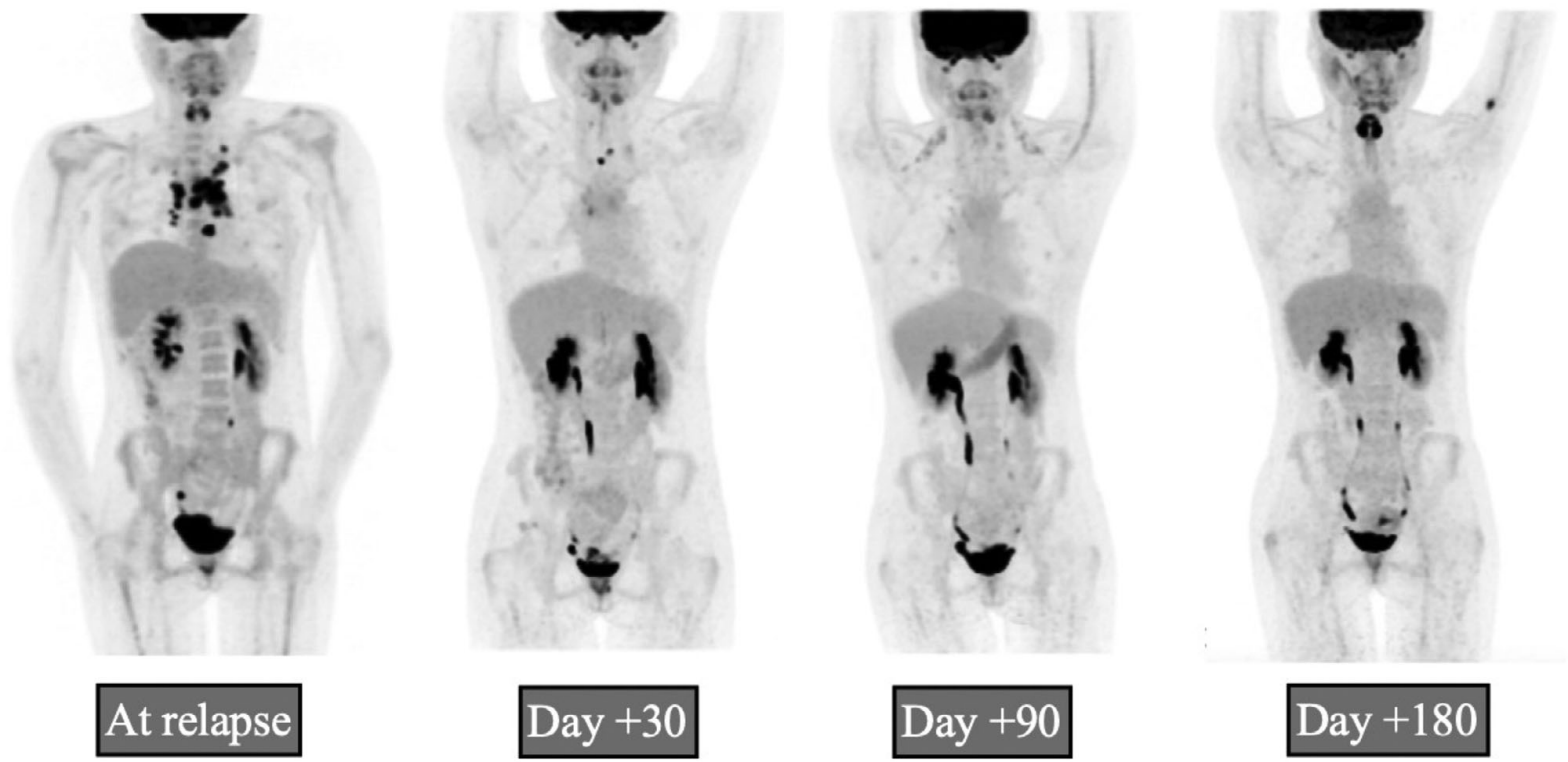
Clinical response at initial presentation and at days+30, +90, and +180 post‐treatment with lisocabtagene maraleucel. Positron emission tomography (PET) computed tomography (CT) scans at these timepoints demonstrate complete response per Lugano Classification lymphoma response criteria.

Per institutional protocol, the patient was closely followed outpatient and evaluated at days +30, +90, and +180 (last day of follow‐up) post‐CAR‐T infusion for response assessment. At day+30, her PET‐CT scan showed CR which persisted at day+90 and day+180 (Figure 2). After discharge, the only significant event was rhinovirus with mild symptoms at day+53 that resolved with supportive therapy. She developed mild cytopenias, specifically anemia and leukopenia with lymphopenia and neutropenia that resolved with daily filgrastim for 3 days at day+89 and did not require transfusion (Figure 1B). B‐cell aplasia with hypogammaglobulinemia persisted until day+180 as commonly seen in patients who underwent CAR‐T [5].

## 3 DISCUSSION

Here we present a case of an adolescent patient with PMBCL who relapsed and was refractory to 3 lines of systemic chemoimmunotherapy and received CAR‐T. To our knowledge, this is the first published case of an adolescent patient receiving liso‐cel.

At the time of evaluation, the patient's disease relapsed within 12 months after first‐line chemotherapy and was refractory to subsequent chemoimmunotherapies including pembrolizumab. Given her disease was chemorefractory, we did not favor high‐dose therapy with autologous hematopoietic stem cell (HCT) as the retrospective studies demonstrated clinical benefit in patients with R/R PBMCL who were chemosensitive or experienced late relapse [4, 6]. Allogeneic HCT was considered as a prospective study that demonstrated durable response in patients with R/R PMBCL who underwent allogeneic HCT [7]. However, this study demonstrated cumulative incidence of non‐relapse mortality and relapse of 32% and 33% at 5 years, and the outcomes were significantly better in patients who responded to prior chemoimmunotherapies than those with refractory disease. Therefore, given the high risk of relapse and complications from allogeneic HCT, we explored alternative therapies such as CAR T‐cell therapy.

While there are multiple CAR‐T products approved for adult patients with R/R large B‐cell lymphoma (LBCL), there are no FDA‐approved CAR‐T products for pediatric and adolescent patients in this setting. Based on published data in adult population, we considered axicabtagene ciloleucel (axi‐cel) and liso‐cel, as these products both demonstrated impressive response and durable response [8, 9]. Given the lower rate of CRS, ICANS, and cytopenia with liso‐cel compared to axi‐cel [8, 9], we selected liso‐cel to minimize toxicity given the patient's young age.

Prior to CAR‐T infusion, the patient received radiation therapy to the mediastinum as bridging therapy. Bridging therapy debulks tumor burden and can enhance tumor microenvironment for optimal response [10, 11]. Our patient tolerated bridging therapy without complications and was able to receive CAR‐T infusion without delay.

The patient tolerated liso‐cel with minimal toxicity. There is one case report describing the experience of a 17‐year‐old female patient who received axi‐cel as the third line of systemic therapy for refractory PMBCL [12]. She received pembrolizumab and radiation to mediastinum as prior lines of therapy and bridging therapy with rituximab and methylprednisolone. After CAR‐T infusion, this patient developed grade 1 CRS at day+4 and grade 3 ICANS at day+5 requiring one dose of tocilizumab and two doses of dexamethasone. In addition, the patient had persistent cytopenias requiring intermittent filgrastim (every 1–6 weeks) and was dependent on transfusion for platelet and red blood cells for 8 weeks post CAR‐T. Our patient did not develop any CRS and ICANS and only needed filgrastim support once without transfusion, suggesting liso‐cel as an alternative safe option for CAR‐T in this population.

Our patient achieved CR at day+30, which persisted through day+180. Similarly, the patient receiving axi‐cel achieved partial response at day+30 followed by CR at day+90 which persisted through 7.5 months. Their successful response is consistent with high rates of response among the adult patients treated with these products [8, 9]. Additionally, adult patients with R/R LBCL who received axi‐cel after at least 2 lines of chemoimmunotherapy demonstrated estimated disease‐specific survival of 51% at 5 years [13]. Given such curative potential of CAR‐T, consolidative allogeneic HCT was not recommended to the patient. Prospective trials of CAR‐T in pediatric and adolescent patients with R/R PMBCL are warranted to confirm these findings.

Another important consideration that arose while treating our patient was emotional distress from unfamiliarity with cellular therapy and its potential side effects. We provided extensive education to both patient and assenting parent and consultation with psychology and spiritual care. Our patient was not interested in having children in the future, but fertility preservation should be discussed with knowledge that there is yet lack of data on the impact of CAR‐T on fertility.

In conclusion, liso‐cel was well‐tolerated and led to CR for 6 months post CAR‐T in an adolescent patient with aggressive PMBCL that relapsed and was refractory to 3 lines of systemic chemoimmunotherapy. The patient did not develop any CRS, ICANS, or significant adverse effects but experienced mild cytopenias requiring filgrastim once at day+89. Psychosocial support and discussion of fertility preservation are important aspects of care for this population. More prospective studies investigating the safety and efficacy of CAR‐T in pediatric and adolescent populations with R/R LBCL are warranted.

## CONFLICT OF INTEREST STATEMENT

Saurabh Dahiya: Advisory board for Kite Pharma. Research funding from Kite/Gilead.

## FUNDING INFORMATION

There is no funding for the case report.

## ETHICS STATEMENT

The authors have confirmed ethical approval statement is not needed for this submission.

## PATIENT CONSENT STATEMENT

Consent was obtained from the patient for this case report.

## CLINICAL TRIAL REGISTRATION

The authors have confirmed clinical trial registration is not needed for this submission.

## Data Availability

The data that support the findings of this study are available from the corresponding author upon reasonable request.
